# Novel image-analytic approach reveals new insights in fine-tuning of slime mould network adaptation

**DOI:** 10.1098/rsos.240950

**Published:** 2024-10-30

**Authors:** Philipp Rosina, Martin Grube

**Affiliations:** ^1^ Institute of Biology, University of Graz, Graz 8010, Austria

**Keywords:** epinephrine, exploration behaviour, image analysis, network complexity, network volume, starvation

## Abstract

This study introduces a novel methodology to explore the network dynamics of *Physarum polycephalum*, an organism celebrated for its remarkable adaptive capabilities. We used two innovative techniques to analyse its growth behaviour and network modifications under stress conditions, including starvation and differential epinephrine exposures. The first method provided a quantitative assessment of growth and exploration over time. The second method provided a detailed examination of vein diameter and contraction patterns, illuminating the physiological adjustments *P. polycephalum* undergoes in response to environmental challenges. By integrating these approaches, we were able to estimate the total network volume of the organism, with a focus on the normalized estimated volume, unveiling insightful aspects of its structural adaptations. While starvation reduced the volume, indicating a significant structural compromise, low and high epinephrine concentrations maintained a volume-to-area ratio comparable with the control. Determining the fractal dimension of the networks over time revealed a fine-tuning of the network complexity in response to environmental conditions, with significant reductions under stress indicating a constrained network adaptation strategy. These methods, novel in their application to *P. polycephalum*, provide a framework for future studies and a basis for exploring complex network behaviours with potential applications in bioengineering and adaptive network design.

## 1. Introduction

*Physarum polycephalum*, a standout model organism within the Myxogastria class, has captivated researchers for over a quarter of a century owing to its sophisticated behaviours and ability to form complex network structures. This brainless slime mould demonstrates capabilities such as navigating mazes [1], optimizing transport networks [2], adapting to stressful substances [3] and modulating its network structures in response to nutrient availability [4]. These behaviours highlight its potential as a bio-mimetic model for understanding network dynamics and decision-making processes in systems lacking central nervous control. The life cycle of *P. polycephalum* is defined by a trophic phase characterized by significant growth and the development of intricate network structures. These structures facilitate periodic cytoplasmic streams crucial for the organism’s adaptation and survival. The ability of *P. polycephalum* to optimize its growth patterns and resource allocation strategies mirrors complex problem-solving behaviours typically associated with higher organisms, suggesting a fundamental commonality in the organizational strategies across life forms.

Recent advancements in molecular biology and genetics have profoundly elucidated Myxomycetes’ ecological roles and distribution patterns, including *P. polycephalum*. Researchers have gained deep insights into their diversity across various habitats by using advanced molecular techniques such as real-time polymerase chain reaction and high-throughput sequencing, thus enhancing our understanding of their ecological adaptations and survival strategies [5]. Experiments have demonstrated that habituated slime moulds can convey adaptive responses to environmental repellents through cell fusion with unhabituated individuals, offering valuable insights into non-neural learning mechanisms and their potential evolutionary significance [6]. Further studies reveal that increased calcium concentrations significantly enhance the growth rate and promote the transition from symmetrical to asymmetrical growth. Three distinct populations have been identified within a clonal lineage, each exhibiting consistent symmetry-breaking behaviours but with different motility characteristics. This underscores the potential for behavioural plasticity to generate cellular diversity crucial for survival [7]. Additionally, it has been observed that slime moulds actively avoid environments previously explored by stressed clone mates, indicating that they exhibit physiological responses to stress that can be sensed by conspecifics [8]. Investigations into the ageing process of slime moulds, aged from 1 to 100 weeks, have shown that while migration speed decreases with age in both favourable and adverse environments, decision-making and learning abilities remain unaffected by age [9]. Moreover, *P. polycephalum* exhibits a unique form of spatial memory that significantly enhances its navigational abilities in highly complex environments. This ability is evidenced by its use of extracellular slime to avoid previously explored areas, facilitating efficient exploration and resource acquisition. This externalized memory not only aids in navigating physical barriers but also implies a primitive form of learning and memory, achieved without the central nervous systems found in more evolved organisms [10]. The impact of environmental stimuli, particularly epinephrine, on the motility of *P. polycephalum* has been a subject of extensive research. Studies have shown how epinephrine can alter the organism’s motility by slowing its movement rates and affecting its behavioural patterns, providing insights into the slime mould’s responsive mechanisms at a cellular level [11]. These findings are further supported by broader research across various biological systems, illustrating how neurotransmitters play a crucial role in the adaptive behaviours of even the simplest organisms [12–15]. Parallel methodological advancements have propelled our ability to quantitatively analyse myxomycete growth and behaviour. The integration of geographical information systems (GIS) software in recent studies has facilitated a more precise assessment of the growth dynamics of these organisms, enabling researchers to make accurate biomass estimations correlated with detailed spatial measurements [16].

In other studies, researchers investigated shear stress and relative vein resistance metrics to understand their roles in vein dynamics within vascular networks of *P. polycephalum*. The findings revealed that shear stress influences vein radius with a notable time delay of 1–3 min, while vein fate is ultimately governed by parameters such as local pressure and relative resistance, which integrate the network’s overall architecture through the conservation of fluid volume [17,18]. Additionally, fractal dimension analysis has become a well-established method to assess the structural complexities and dynamic patterns of network formation in *P. polycephalum*. This approach allows researchers to quantify the complexity within the organism’s network structure, providing a deeper understanding of how environmental conditions influence morphological adaptations [19–22]. Recent structural analyses using advanced imaging techniques such as scanning electron microscopy and transmission electron microscopy have revealed the detailed architecture of *P. polycephalum*’s plasma membrane pores and internal channel systems. These studies have shown that the organism’s morphological adaptations, specifically its ability to form micro-, meso- and macroplasmodia, are intricately linked to its functional requirements, facilitating efficient navigation and resource distribution within complex environments [23]. As the body of research grows, exploring contraction patterns and the underlying mechano-bio-chemical feedback mechanisms within *P. polycephalum* continues to provide invaluable insights. These studies demonstrate that confined plasmodia exhibit various contraction patterns critical for locomotion, supported by gel and sol phases within their structures, which are typical of mature plasmodia [24]. In summary, the diverse studies on *P. polycephalum* enhance our understanding of this fascinating organism and contribute to broader scientific inquiries into the principles of network dynamics, adaptive behaviour and survival strategies across biological systems. However, traditional methodologies often require high-cost equipment and may not precisely capture the organism’s dynamic adaptations. Our research addresses these challenges by introducing innovative analytical techniques that use a simple, cost-effective set-up with high temporal and spatial resolution. This approach enables a comprehensive exploration of how *P. polycephalum* adapts its network behaviour in response to environmental stresses, positioning it as a prime model for bioengineering and complex system studies.

## 2. Results

**Figure 1.**
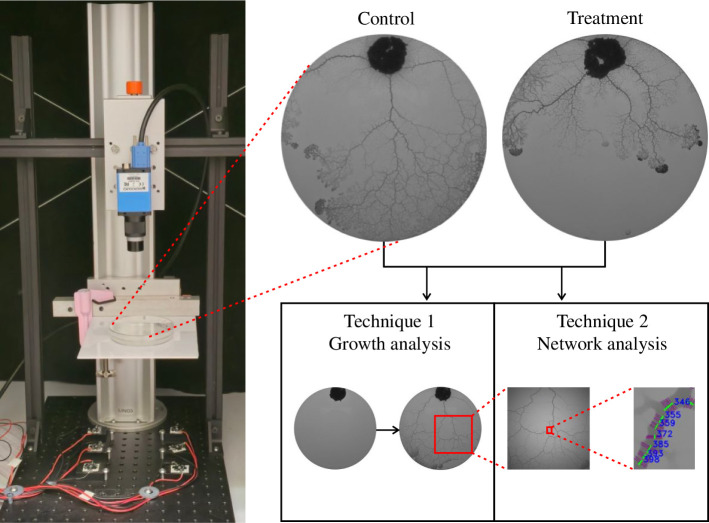
Comprehensive overview of methodological approaches and camera recording settings. This figure presents a detailed schematic representation of the experimental set-up used to capture the dynamic growth and physiological responses of *P. polycephalum*. It showcases the camera settings and recording intervals used to monitor the organism’s behaviour over time. The figure also includes comparative images highlighting the distinct visual differences between control conditions and epinephrine treatments. Additionally, a schematic diagram summarizes the principles behind the two novel, low-cost techniques developed in this study: one for high-resolution growth and exploration analysis and the other for detailed microscopic examination of vein diameter and contraction patterns. These techniques, which require minimal resources, provide unprecedented insights into the adaptive strategies of *P. polycephalum* under various environmental stress conditions.

Before presenting the findings of our study, it is essential to delineate the methodologies underlying our analysis. We employed two novel techniques to investigate the network properties of *P. polycephalum*, each designed to capture distinct aspects of its growth and adaptive behaviours. The first technique provides a quantitative assessment of growth over time and the capacity for environmental exploration. This method allows for a dynamic view of *P. polycephalum*’s expansion and resource-seeking strategies, offering insights into its spatial and temporal adaptation mechanisms. The second technique offers a detailed examination of vein diameter and contraction patterns across hundreds of positions in the network, providing a comprehensive physiological snapshot of how the organism responds to environmental stimuli. This approach enhances our understanding of slime mould’s internal signalling and structural adaptation and complements the first technique by contributing to a comprehensive view of its network dynamics (figure 1).

### 2.1. Measured growth, recorded exploration and complexity changes of *Physarum polycephalum* in response to environmental and chemical stimuli over 24 hours

**Figure 2.**
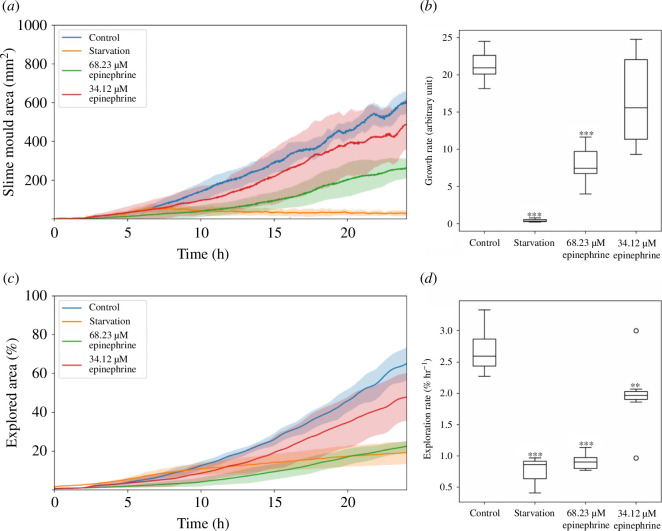
Analysis of growth and exploration dynamics of *P. polycephalum* under various conditions. This figure comprehensively overviews the organism’s response to control, starvation and different epinephrine concentrations. (*a*) A line plot illustrating the two-dimensional biomass accumulation over time, highlighting the differential growth patterns under each condition. (*b*) Detailing the growth rates, comparing the statistical distribution between the control, starvation and epinephrine-treated groups to elucidate the impact on growth velocity. (*c*) The plot showing the extent of environmental exploration over time demonstrates the organism’s adaptive exploration behaviour. (*d*) The exploration rates provide a quantitative comparison of how each condition influences the rate at which *P. polycephalum* explores its surroundings. The shaded areas in (*a*) and (*c*) demonstrate the standard deviation (*σ*), effectively highlighting the variability among replicates. Statistical significance is indicated by asterisks: ** (*p* ≤ 0.01), and *** (*p* ≤ 0.001).

**Figure 3.**
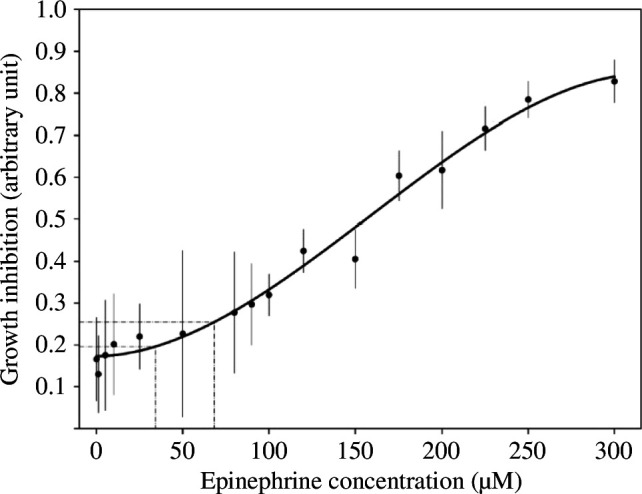
Dose–response curve of growth inhibition in *P. polycephalum*. This graph illustrates the relationship between epinephrine concentration (µM) and growth inhibition of *P. polycephalum*. Growth inhibition is quantified as the normalized difference in the covered area relative to the control, calculated as ${Area}_{c}-AVG\left({Area}_{c0}\right)/AVG\left({Area}_{c0}\right)$ , where Area_*c*_ is the covered area at each concentration and Area_*c*0_ is the average covered area at zero concentration. The curve represents a fitted polynomial function degree 3 to the experimental data points, with error bars indicating standard deviations, underscoring the variability observed across multiple trials.

Our findings reveal distinct responses to differing epinephrine concentrations and starvation in examining the impact of various conditions on the growth of *P. polycephalum*. Specifically, exposure to a low concentration of epinephrine significantly inhibited growth compared with the control, underscoring a sensitive reaction even at minimal chemical stimuli (figure 2*a*). Interestingly, both the high concentration of epinephrine and the starvation condition resulted in similarly pronounced reductions in growth, with both conditions exhibiting growth rates significantly lower than those observed in the control group. A detailed analysis of growth rates further highlights the sheer contrast under these experimental conditions. The growth rate in conditions of starvation was approximately 20 times lower than in the control, illustrating the profound impact of nutrient deprivation on the expansion of the organism. Conversely, while the high concentration of epinephrine led to a significant decrease in growth rate (figure 2*b*), the lower concentration of epinephrine showed a less pronounced effect, which did not differ significantly from the control. This gradation in response is illustrated by the dose–response curve (figure 3), indicating a proportional relationship between epinephrine concentration and growth inhibition in *P. polycephalum*.

Our investigation into the exploration dynamics of *P. polycephalum* under varying conditions revealed a universal decrease in the extent of explored area and exploration rate compared with the control, with all tested conditions resulting in significantly reduced exploration (figure 2*c*,*d*). Notably, there was no significant difference between the exploration under starvation and high epinephrine concentration conditions, suggesting a similar limitation on the organism’s ability to explore its environment in these scenarios. This finding is particularly intriguing when considering the growth data, where the high epinephrine concentration condition still permitted some growth, albeit at a reduced rate. The minimal exploration observed in this context suggests that the network does so slowly and compactly while growing. This contrast underscores a distinct adaptive strategy of *P. polycephalum*. Under certain stress conditions, it appears to focus on maintaining or expanding its network within the constraints imposed by the environment. This strategy may reflect an optimization of the organism’s response, enhancing its ability to survive in less favourable conditions.

**Figure 4.**
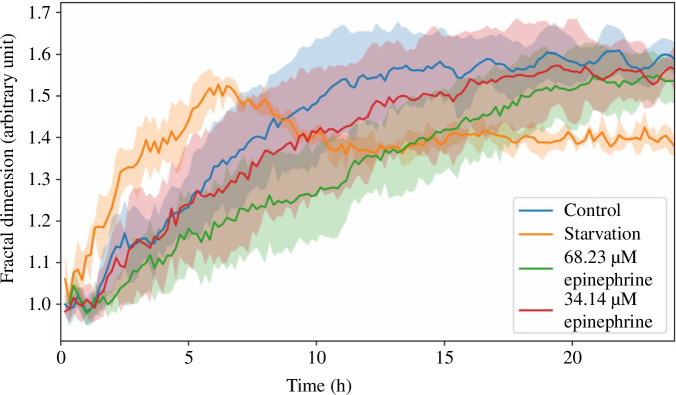
Temporal evolution of fractal dimension in *P. polycephalum* under varying conditions. This figure displays the changes in fractal dimension over 24 h for *P. polycephalum* subjected to different experimental conditions: control (blue line), starvation (orange line) and epinephrine treatments at concentrations of 68.23 µM (green line) and 34.14 µM (red line). The fractal dimension was calculated from a selected subset of images, specifically one image every 10 min, taken from the comprehensive image set captured from time point 0 to 24 h. This subset illustrates the organism’s network complexity as it adapts to environmental and chemical stimuli. Shaded areas represent the standard deviation, indicating the variability among replicates. The plot reveals how stress conditions influence the structural complexity of the slime mould’s network, with lower fractal dimensions under high stress suggesting constrained network complexity.

Figure 4 displays the evolution of fractal dimensions in *P. polycephalum* over 24 h under varied conditions, elucidating how the organism’s network complexity responds to environmental and chemical stresses. The control condition consistently rises in fractal dimension, indicating an ongoing increase in network complexity. This steady enhancement suggests robust network expansion and adaptability in standard growth conditions. Contrastingly, networks subjected to starvation show a distinct pattern: a sharp increase in complexity within the first 6 h, surpassing the control’s growth in complexity, followed by a significant decrease, stabilizing at a fractal dimension around 1.4. This behaviour implies an initial robust expansion, possibly as a rapid adaptive response to nutrient scarcity, which later transitions into a simplified or stable state as resources are depleted or the organism adjusts to the stress. In response to epinephrine, the dynamics vary by concentration. The lower concentration (34.14 µM) closely tracks the control, suggesting that minor chemical stress does not substantially alter the organism’s ability to develop its network complexity. However, the higher concentration (68.23 µM) results in a slower increase in complexity, eventually converging with the control and lower concentration levels by the end of the observation period. This pattern might indicate a delayed but eventual adaptation to the higher chemical stress, potentially involving acclimation mechanisms that allow the organism to maintain functional network integrity under sustained exposure.

### 2.2. Structural and physiological responses: analysing vein diameter and contraction frequency

**Figure 5.**
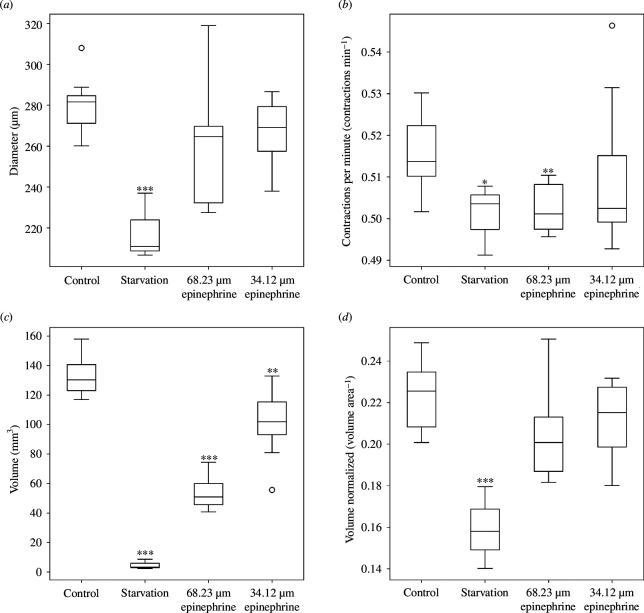
Physiological and structural responses of *P. polycephalum* across experimental conditions. This figure presents the variations in (*a*) vein diameter and (*b*) contraction frequency, which were analysed using the second set of images captured from hour 24 to hour 25 at a frequency of one image every 4 s. Plot (*c*) shows the estimated network volume, and (*d*) displays the normalized estimated volume. Both (*c*) and (*d*) use a combined approach, integrating results from the initial growth analysis and the vein diameter measurements detailed in (*a*) These metrics collectively offer insights into the intricate balance between structural integrity, physiological activity and growth efficiency in response to environmental and chemical stimuli, illustrating how *P. polycephalum* adjusts its physical structure and functional capabilities under varying conditions. Statistical significance is indicated by asterisks: * (*p* ≤ 0.05), ** (*p* ≤ 0.01), and *** (*p* ≤ 0.001).

In our analysis of vein diameter and contraction frequency in *P. polycephalum*, distinct responses to environmental and chemical stimuli were observed. Notably, the vein diameter was significantly reduced only under starvation conditions, suggesting a pronounced structural adaptation to nutrient scarcity. By contrast, treatment with epinephrine, regardless of concentration, did not elicit a significant change in vein diameter, indicating that the structural integrity of the network’s veins remains largely unaffected by these chemical stimuli (figure 5*a*). The analysis of contraction frequency revealed a more nuanced response. Both starvation and high epinephrine concentrations significantly reduced averaged contraction frequency, highlighting a decrease in the organism’s physiological activity under these stressors. Interestingly, the low concentration of epinephrine did not affect the averaged contraction frequency, suggesting a threshold below which *P. polycephalum*’s pulsatile activity remains stable (figure 5*b*). When directly comparing the effects of starvation with high concentrations of epinephrine on contraction frequency, a *t*‐test confirmed no significant difference between these conditions, indicating a similar level of physiological response to these distinct types of stress. However, the comparison of their effects on vein diameter approached significance, with a *p*-value of 0.052, suggesting a marginal difference (figure 5*a*) that might indicate subtle physiological distinctions not fully captured by this analysis. This near-significant result points to the complexity of *P. polycephalum*’s adaptive mechanisms, where even closely related stress responses may manifest differently at the structural level.

### 2.3. Estimated network volume in *Physarum polycephalum*: an analysis using combined methodological approaches

Using the combined results from the two techniques, we have developed a comprehensive method to calculate the estimated network volume of *P. polycephalum*, providing a unique insight into its adaptive responses under varying environmental and chemical conditions. This integrative approach allows a nuanced understanding of how the organism modulates its physical structure in response to stress. Our analysis revealed that the estimated network volume is significantly impacted under stress conditions. Precisely, a pronounced reduction in volume under starvation conditions reflects a substantial decrease in the capacity of the organism to maintain or expand its network when deprived of nutrients. Similarly, exposure to a high concentration of epinephrine resulted in a network volume reduction to approximately half of the control, indicating a significant impact of this chemical stressor on *P. polycephalum*’s structural adaptation. The estimated network volume at a low concentration of epinephrine was also reduced, albeit to a lesser extent, which aligns with observations of a diminished network size under these conditions (figure 5*c*). We observed a pattern when normalizing these results to the two-dimensional biomass area at 24 h. Starvation conditions led to a significantly reduced network volume, probably owing to the decreased vein diameter observed under this condition. Conversely, neither concentration of epinephrine showed a significant difference from the control when normalized in this manner. This suggests that, despite the reduction in estimated network volume, the organism maintains a relatively consistent volume-to-area ratio under epinephrine exposure, contrasting with the sheer reduction observed under starvation (figure 5*d*).

## 3. Discussion

This study introduced two innovative techniques to dissect the network properties of *P. polycephalum*, providing a detailed view of its adaptive responses to environmental and chemical stimuli. Our findings reveal a nuanced strategy for survival and growth in response to nutrient scarcity and epinephrine exposure, building upon and extending foundational insights from earlier studies. The dose–response curve, which quantitatively assessed the impact of varying epinephrine concentrations, demonstrated a significant growth inhibition under both low and high concentrations, reinforcing the observations from 1977 that first documented epinephrine’s impact on *P. polycephalum*’s motility [11]. This inhibition pattern underlines the sensitive modulation of growth processes in response to epinephrine, adding a quantitative layer to the qualitative observations made in previous decades. The stark contrast in growth rates under starvation, 20 times lower than the control, underscores the critical importance of nutrient availability. This aligns with broader implications of neurotransmitter interactions observed in other biological systems, including the innate immune response [25]. Furthermore, our detailed analysis of vein diameter and contraction frequency, enhanced by the fractal dimension analysis, offers new insights into the physiological adaptations of *P. polycephalum*. The fractal dimension analysis, in particular, provided a unique perspective on the organism’s structural complexity, revealing how it dynamically adjusts its network in complex environments. The reduction in vein diameter exclusively under starvation conditions suggests a structural economization that directly contributes to the organism’s survival strategy, complementing and expanding our understanding of *P. polycephalum*’s adaptive mechanisms. The stability of vein diameter in the presence of epinephrine, regardless of concentration, indicates the resilience of the structural framework against chemical stress, provided that nutrients are not limited. This observation offers a finer resolution to the complex interplay between structural integrity and physiological activity under varying stress conditions, further illuminating the organism’s multifaceted response strategies. Our approach to estimating network volume, particularly focusing on the normalized volume-to-area ratio, reveals the organism’s sophisticated balance between network expansion and density under stress. The introduction of error estimation plots has allowed us to rigorously assess the potential inaccuracies in our volume measurements owing to network geometry assumptions, thus enhancing our findings’ reliability. The ability of *P. polycephalum* to maintain a similar volume-to-area ratio under epinephrine exposure, similar to the control condition, demonstrates an efficient modulation of network volume, ensuring its survival and adaptation. This trait highlights the organism’s resilience and suggests potential parallels with other biological systems where network dynamics play a crucial role in organismal adaptation, as hinted at by advancements in growth measurement techniques using GIS software [16]. As detailed in our study, the adaptive responses of *P. polycephalum* to starvation and epinephrine exposure contribute significantly to the broader understanding of adaptive network dynamics in biological systems. By elucidating the complex interplay between growth, exploration and network volume adjustments in response to external stimuli, our research positions *P. polycephalum* as a model organism for exploring the resilience and flexibility of biological networks. However, several limitations should be noted. Our experimental set-up, while controlled, may not fully replicate the environmental variability present in natural conditions, potentially constraining the organism’s adaptive responses. Additionally, the volume estimation model relies on simplified geometric assumptions that may not entirely capture the true complexity and irregularity of the network morphology. Similarly, while the fractal dimension analysis provides valuable insights into network complexity, it may not encompass all facets of the organism’s structural adaptations. Future research could further deepen our understanding of the molecular mechanisms underlying these adaptive strategies by integrating more sophisticated imaging techniques and refined modelling approaches. Such advancements could yield broader implications for bioengineering, network theory and adaptive system design, providing a robust framework for exploring the universal principles governing biological adaptability.

## 4. Methods

### 4.1. Cultivation and sample preparation

*Physarum polycephalum* was obtained from Leicester, United Kingdom (no. LU352). The organism was meticulously cultured in sterile Petri dishes (Sarstedt, 92 × 16 mm, no. 82.1473.001), employing a growth medium composed of 1.2 % agar-agar (Roth, Agar-Agar Kobe I, no. 5210) prepared with autoclaved milliQ-H_2_O (Milli-Q IQ7000, Merck). To simulate a controlled and consistent environment, the cultures were maintained in a darkened incubator with an optimal temperature range of 20 to 22 °C to reach the ideal growth rate and morphological development of *P. polycephalum*. The slime moulds were nourished with autoclaved extra soft full grain oat flakes (‘Kölln Flocken’, Peter Kölln GmbH & Co. KGaA, Germany), selected for their high nutrient content and suitability for sustaining healthy plasmodial growth. To ensure the vitality and genetic consistency of the cultures, a segment of the plasmodium, along with a portion of the oat flakes, was subcultured to a fresh agar plate every three days. This routine was strictly adhered to, preventing nutrient depletion and contamination, and promoting the cultures’ longevity. A circular piece, with a diameter of 15 mm, comprising a mixture of oat flakes and *P. polycephalum* was transferred for the control and both epinephrine conditions. By contrast, a piece of the same size was used for the starvation condition, containing the slime mould network without oat flakes to simulate nutrient deprivation. The Petri dishes were prepared with a base layer of 1.2 % agar-milliQ-H_2_O, serving for the control and starvation condition as medium. For the stress experiments, the medium was supplemented with 1 : 80 000 (68.23 µM) or 1 : 1 75 000 (34.12 µM) (-)-epinephrine (Sigma, no. E4250). These concentrations were chosen based on preliminary studies (figure 3), with the highest concentration selected to achieve close to 0.25 growth inhibition, ensuring a measurable stress response in *P. polycephalum*. The lower concentration was set at around 0.2 growth inhibition to observe a borderline effect, which marks where the dose–response curve rises. Unlike methods described in other publications [11], where epinephrine is applied directly via drops or injections, in our set-up, epinephrine was incorporated into the agar medium. This method ensures a consistent presence of the compound, starting from the transfer of the organism to the experimental Petri dish and continuing throughout the duration of the experiments, allowing for diffusion and active uptake by the organism. All procedures, from preparation of media to transfer of plasmodium segments, were conducted under aseptic conditions to prevent microbial contamination and ensure the integrity of the experimental results.

### 4.2. Image recording and processing

Our image recording set-up, illustrated in figure 1, was placed in a temperature-controlled dark room, maintained at 20 to 22 °C to mirror the cultivation conditions of *P. polycephalum*. This setting ensured minimal external influence on the organism’s behaviour during recording. We used an industrial-grade colour camera, the DFK 38UX541, and a TMN 0.3/110 macro lens, both obtained from ‘The Imaging Source Europe GmbH’. For the initial phase of growth recording, the Fujifilm lens HF12.5Sa-1 was employed, chosen for its high-resolution imaging capabilities. For the photo recording process, green LEDs were employed based on publications [26,27] indicating that green light does not influence *P. polycephalum*’s behaviour, in contrast to white or other colours of light, which can affect its growth and movement patterns. These LEDs were interfaced with an Arduino Uno Rev3, ensuring precise control over the lighting during image acquisition. Both the Arduino and the camera were connected to a Raspberry Pi 4B, which served as the central control unit for the recording set-up. The Raspberry Pi operated on Raspberry Pi OS 64-bit, providing a stable and efficient platform for image capture. The image capture process was initiated following the transfer of a 15 mm circular slime mould sample. A custom ‘recording Python (V3.10) script’ (including files from The Imaging Source Company GitHub—‘tiscamera’) was developed to automate the image recording. For the first 24 h, the script was programmed to capture one image every 60 s using the Fujifilm lens. These images were used to assess the growth over time, growth rate, exploration and exploration rate of *P. polycephalum*. A subset of these images, precisely one every 10 min, was used to conduct the complexity analysis through fractal dimension calculation. Following this period, we switched to the macro lens to obtain closer, more detailed network images at a higher frequency—every 4 s for an hour from the 24th to the 25th hour. This image set is used for vein diameter measurements and frequency analysis of the contraction patterns. During each capture event, the green LEDs were briefly activated for less than half a second to minimize disturbance to the organism. Camera settings were meticulously optimized for clarity and detail, with an exposure time of 55 555 µs, a gain of 23.7, a black level of 240 and a gamma setting of 1. An ‘image processing Python script’, using the noise reduction capabilities of the OpenCV (V4.5.5) library (as detailed in table 1), was employed for this purpose. This script was designed to automatically load, denoise and save each processed image, streamlining the workflow and ensuring consistency in image quality across all samples.

**Table 1.** Overview of Python functions and parameters used in image processing, growth and network analysis. (This table summarizes the specific Python functions and their corresponding parameters employed in the image processing and analysis steps of our study).

**denoising: OpenCV—fastNlMeansDenoising**			
filter strength: 8	template window: 5	window: 21	
**growth algorithm: OpenCV—adaptive threshold**			
cv2.ADAPTIVE_THRESH_GAUSSIAN_C	threshold type: cv2.THRESH_BINARY_INV	window: 71	tune: 4
**vein contraction algorithm: OpenCV—adaptive threshold**			
cv2.ADAPTIVE_THRESH_GAUSSIAN_C	threshold type: cv2.THRESH_BINARY_INV	window: 69–91	tune: 2
**circle mask detection: OpenCV—HoughCircles**			
method: cv2.HOUGH_GRADIENT	minDist: 563	param1: 100	Dp: 1
minRadius: 1500	maxRadius: 1750	param2: 30	
**section detection: OpenCV—HoughLinesP**			
threshold: 25	min line length: 10	theta: π/180	rho: 1

### 4.3. Methodological approaches for analysing growth and exploration

To accurately analyse the growth of *P. polycephalum*, it was imperative to focus solely on the relevant growth area. We achieved this by implementing a mask that limited our region of interest (ROI) to the growth region within the Petri dish. The ROI determination was crucial for excluding extraneous data outside the inner circle of the dish. The mask was automatically generated using the cv2.HoughCircles function from the OpenCV library (details in table 1), which detects the circular boundary of the Petri dish. Once the ROI was established, the image analysis algorithm loaded each image in greyscale. This conversion simplifies the data and enhances the contrast, facilitating more effective analysis. The algorithm then applies the previously defined mask to each image, ensuring that only the area within the Petri dish is analysed. Following masking, the images undergo thresholding using the adaptive threshold method from OpenCV (referenced in table 1). This step is pivotal as it segregates the slime mould network from the background, allowing for precise pixel-based analysis. Growth is measured by the increase in the two-dimensional area of the slime mould, reflecting natural physical expansion. The foundation of our growth analysis was calculating the pixel count corresponding to the slime mould network in each image. This pixel count is a proxy for the slime mould’s area at each time point. A metric scale was implemented by using a reference image with a known size, which allowed the unit to be changed from pixels into square millimetres (mm^2^). We normalized the data by subtracting the area measurement of the initial time step (t_0_) from all subsequent measurements to represent growth over time accurately (figure 2*a*). This normalization process accounts for any initial size variations and focuses on the actual growth increment. The growth rate was then calculated using equation (4.1), which quantifies the slime mould network’s expansion rate over the observed time.

In our study, obtaining exploration data for *P. polycephalum* was a meticulous process. The position of the slime mould was recorded with great precision in each image, corresponding to specific time points throughout the experiment. This systematic tracking, enabled by our novel set-up, allowed for a precise calculation of the area explored by the slime mould (*EA*_*t*_) at each time *t*. This technique provides high temporal resolution data on growth and exploration dynamics, offering an accessible alternative to more complex and costly traditional methods. The exploration tracks each position reached within the Petri dish up to a given time point. A stable level of growth (no area increase) combined with an increase in exploration over time would indicate movement rather than growth. The exploration rate is calculated with equation (4.2). A comprehensive table of symbols used throughout the manuscript can be found in table 2, providing clear definitions and facilitating an easier understanding of the mathematical notations employed in this study.

**Table 2.** Definitions of symbols used in the study.

symbol	description	equation/figure
Area_*c*_	area at concentration *c*	Figure 3
Area*_c_*_0_	area at concentration *c* = 0	Figure 3
*A_t_*_24_	area at the time point 24 h	equation (4.1)
*A_t_*_0_	area at the time point 0 h	equation (4.1)
*EA_t_*_24_	explored area at the time point 24 h	equation (4.2)
*F_s_*	sampling frequency	equation (4.3)
*N*	total number of points	equation (4.3)
*p*	probability	equation (4.5)
*r*	radius	equation (4.6)
*l*	length	equation (4.6)
*ε*_*S*_	possible error—circular segment cross section	equation (4.7)
*A*_*C*_	circular cross-section area	equation (4.7)
*A*_*S*_	circular segment cross-section area	equation (4.7)
*ε*_*E*_	possible error—elliptical cross section	equation (4.8)
*A*_*E*_	elliptical cross-section area	equation (4.8)


(4.1) $$growth\;rate=\;\frac{A_{t24}-A_{t0}}{A_{t0}},$$

(4.2) $$exploration\;rate=\;\frac{EA_{t24}}{24}.$$

### 4.4. Complexity analysis

In this study, we aimed to quantitatively assess the complexity of the network structures formed by *P. polycephalum* under various experimental conditions. To achieve this, we analysed the network’s fractal dimension [28,29] over time, using a subset of images from the first technique focused on growth analysis (figure 1). To ensure the efficiency and reliability of our analysis, we optimized our method by selecting one image every 10 min from the high-frequency image capture (one image per minute). This reduced frequency was sufficient to capture the essential dynamics of the network development without compromising the resolution of our temporal analysis. The selected images were then processed to extract the network’s structure, which is the basis for calculating the fractal dimension. The fractal dimension, a scalar number that indicates how a fractal appears to fill space as one zooms down to finer scales, is a key tool in our complexity analysis. It provides a single metric to describe the growing complexity of the slime mould’s network over time. We calculated the fractal dimension using a box-counting method [29] implemented in Python. Analysing the fractal dimension of *P. polycephalum* networks is scientifically intriguing because it provides insights into the adaptive strategies of the organism under different environmental conditions. As the network structure becomes more complex, its fractal dimension increases, reflecting a more efficient spatial exploration and resource allocation capability. This analysis allows us to infer the underlying biological processes driving network formation and adaptation in response to environmental stresses, contributing to a deeper understanding of pattern formation and optimization in biological systems.

### 4.5. Network analysis

**Figure 6.**
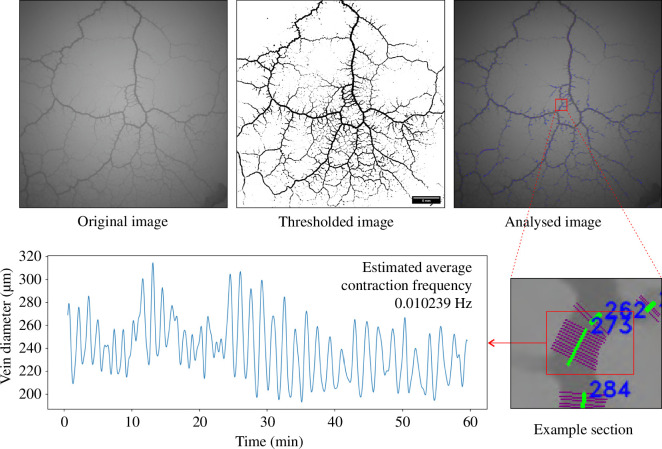
Illustration of critical methodological steps and a representative result example. This figure provides a visual summary of essential stages in the technique, from initial set-up to data acquisition and processing. It details the sequence of critical actions undertaken to ensure the precision and reliability of our experimental approach. Additionally, the figure includes an example of a result obtained using this method, showcasing the direct application of these steps in generating meaningful data.

For an easier understanding, we prepared an overview figure that highlights some of the critical steps involved in this technique, illustrating the key methodologies and their implementation (figure 6).

The fundamental step of our network analysis involved precise position detection on the slime mould network. To delineate the network, the algorithm initially applies the OpenCV adaptive threshold function to the first image (table 1). This thresholded image is then skeletonized iteratively until a single-pixel-wide skeleton remains in the centre of each vein. Potential measuring positions along this skeleton are identified using the cv2.HoughLinesP function (table 1). For each detected segment, the algorithm evaluates the greyscale value at specific positions and extends along the normal vector of the detection line, until the background colour is reached. These positions are systematically analysed, with at least four measurements per segment, depending on the length of the detection line. Given the initial high count of detected sections, rigorous quality control steps were implemented to ensure accuracy: very rough criteria were initially implemented to remove all absolutely wrong positions, and later, after the whole image dataset was processed, finer criteria were used to decide the accuracy of a measured position:

- initial size filtering: sections averaging over 1 mm are discarded after the first image is measured, as these erroneously large measurements do not represent real slime mould veins;
- positional consistency check: the algorithm assesses the uniformity of measurement lines within each section. Sections at vein intersections or with significantly divergent measurements are scrutinized for outliers and adjusted or removed as necessary;
- median comparison: the median size of each section is compared with the overall median. Sections with medians twice the overall are excluded;
- colour gradient analysis: sections failing to distinguish between vein and background are removed; and
- overlap elimination: overlapping sections are identified, with redundant ones being discarded.

All positions failing to meet these stringent criteria will be excluded. Each active section position is saved and measured in each image over time, yielding temporal data on vein size. To mitigate background noise, a fast Fourier transformation (FFT) followed by a power spectral density (PSD) calculation is employed. The 1.5× interquartile range (1.5×IQR) is used to identify the borders between signal peaks and noise. This information creates a filter for the FFT data to remove the noise. An inverse FFT is performed after the filter is applied to the FFT data to obtain the denoised contraction wave data. Observations indicated variable contraction frequencies over time, which posed challenges for applying the FFT directly over 1 h intervals owing to the non-stationarity of the data. To effectively address this issue, we employed a modified Welch’s method. This modified approach enhances the PSD estimation when the signal frequency is not constant over time. Welch’s method segments the data into smaller, overlapping slices, specifically 90 % overlap in our study, to ensure that within each segment, the frequency characteristics are relatively stationary and representative of that specific interval. Each segment then undergoes FFT analysis using a standard Hanning window, which helps to minimize spectral leakage and edge effects, crucial for maintaining accuracy when the signal contains discontinuities or sharp transitions. The PSD is computed for each slice, allowing for a detailed spectral analysis that is more robust to variations within the signal.

The estimated contraction frequency for each slice is precisely determined using equation (4.3), as outlined in Application Note 041 by National Instruments [30]. *N* represents the total number of points in the acquired time-domain signal, while *F*_s_ denotes the sampling frequency, indicating the rate at which the time-domain signal was sampled to convert it from a continuous to a discrete form. The average of these segmental values then represents the overall estimated contraction frequency for the observation period.


(4.3) $$est.\;frequency=\;\frac{\sum_{i=j-3}^{j+3}\left({power}_{i}*i*\frac{F_{S}}{N}\right)}{\sum_{i=j-3}^{j+3}{power}_{i}}.$$

A concluding quality check using the 1.5×IQR method identifies potential outlier sections. The final vetted data were exported as CSV files for subsequent statistical analysis. figure 6 shows an example of the contraction pattern of one single position. This approach uses our novel image analysis techniques, which, unlike traditional methods, do not rely on invasive procedures or high-cost imaging equipment. We captured vein diameter and contraction patterns with high precision across hundreds of positions using a simple camera set-up and Python scripts for automation. This method is advantageous because traditional FFT methods assume that the signal’s frequency components are stable throughout the recording. However, physiological responses can vary significantly in biological systems like *P. polycephalum*, making Welch’s method more suitable for capturing the true dynamical nature of the contraction frequencies. The estimated frequency was measured at each position, and this signal was averaged over the whole organism, as our study focuses on comparing different individuals/conditions rather than between various positions. Our approach demonstrates that it is possible to achieve detailed physiological measurements using cost-effective and accessible techniques, thus broadening the potential for future research in similar biological systems.

### 4.6. Volume estimation methodology for *Physarum polycephalum*: a comprehensive approach

**Figure 7.**
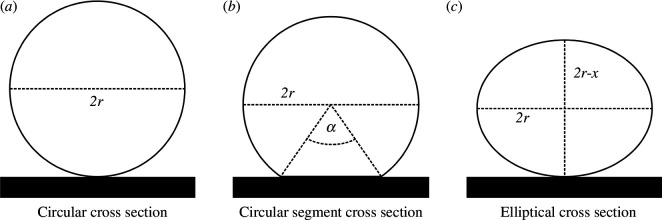
Variations in cross-sectional profiles of the *P. polycephalum* network. This figure depicts three distinct possibilities for the cross-sectional shape of a *P. polycephalum* network. (*a*) showcases a perfectly circular cross section, representing an idealized, symmetrical distribution. (*b*) illustrates a circular segment cross section, indicating a deviation from perfect symmetry owing to environmental or growth-related factors. (*c*) presents an elliptical cross section, demonstrating further variation in network structure potentially influenced by external pressures or internal dynamics. These representations highlight the morphological diversity within *P. polycephalum* networks and underscore the complexity of accurately estimating network volume. All shapes have the same horizontal diameter denoted as *2r* (2× radius).

To aid in the comprehension of our volume estimation model and the associated error calculations, we have included a schematic figure depicting the three potential network cross-sections considered in our study: circular (figure 7*a*), circular segment (figure 7*b*) and elliptical (figure 7*c*).

The core of our volume estimation approach revolves around treating the *P. polycephalum* network as a series of circular sections. The volume estimation model is built on the premise that the entire network can be divided into discrete sections, each characterized by its diameter (2*r*). Our network analysis algorithm measured the diameter at hundreds of positions across the network, providing a dataset of diameters sorted from minimum to maximum. To manage this data, we divided it into intervals based on diameter size, determining the step size for each interval using equation (4.4) ( $\Delta \;veindiamter=max_{veindiameter}-\;min_{veindiameter}$ ). For instance, if the minimum vein size is 200 µm, the first interval would range from 200 µm to 200 µm + stepsize. For our analysis, we decided to use 50 intervals (*N*). For each interval, we calculated the proportion of measurements falling within it. This proportion (*p*) was obtained by dividing the number of measurements in the interval by the total number of measurements.

This step helps us understand the prominence of each interval in the overall network


(4.4) $$stepsize=\;\frac{\Delta_{veindiamter}}{N}.$$

Using the area measurement (in mm^2^ at the 24 h time point from our growth analysis algorithm, which represents the entire network size, we estimated the area represented by each interval. 2*r* represents the centre of the interval and was used to determine the section’s length for that interval using equation (4.5). This length estimation is crucial as it represents the length of the network corresponding to the specific diameter interval:


(4.5) $$l=\;\frac{area*p}{2r}$$

**Figure 8.**
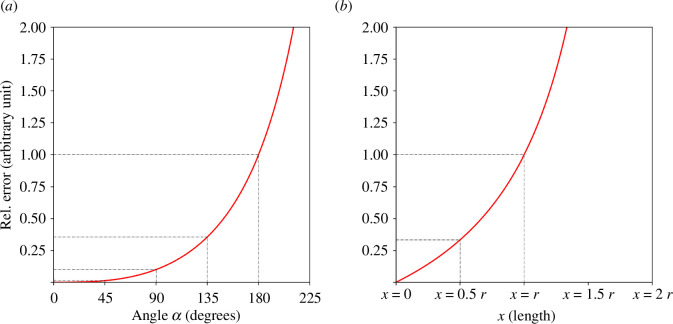
Relative error estimation in volume calculations for different network geometries. This figure demonstrates the estimated relative errors in volume calculations for *P. polycephalum* networks, assuming two non-ideal geometries: a network with a circular segment cross section (*a*) or an elliptical cross section (*b*). Plot (*a*) shows the relative error (equation (4.7)) for a circular segment cross-section shape with varying angles (*α*), plotted against the relative error. The right plot (*b*) depicts the relative error (equation (4.8)) for an elliptical cross-section network, where *x* varies from a circular cross section (*x* = 0) to an increasingly narrower ellipse. The red lines in both plots illustrate how deviations from a perfect circular cross-section shape affect the volume estimates, with *α* and *x* serving as parameters that define the geometry of the network’s cross section.


The total estimated volume of the entire network is calculated by summing the volumes of all individual sections, with the volume of each section derived from its length and average diameter (equation (4.6)). Recognizing the potential discrepancy owing to the assumption of a circular network, we also derived equations to estimate the possible error (*ε*) if the network assumes other shapes, specifically circular segment cross section (equation (4.7)) or elliptical cross section (equation (4.8)). *A*_C_ represents the cross-section area of the circular system, *A*_S_ is the cross-section area of the circular segment system and *A*_E_ is the area of the elliptical cross section. These error estimation equations account for the variations in network shape and offer a range of possible volumes, providing a more comprehensive understanding of the network’s volumetric properties. Figure 8 illustrates the relative error in volume estimations as a function of varying shape parameters *α* and *x*, which define the deviation from the ideal circular cross section to more complex geometries such as circular segment and elliptical structures. This plot enables a more accurate assessment of potential discrepancies in volume calculations, aiding in refining our estimates based on observed network geometries.

(4.6) $$est.vol=\;\sum_{n=1}^{N}r_{n}^{2}\pi *l_{n},$$

(4.7) $$\varepsilon_{S}=\frac{A_{C}-A_{S}}{A_{S}}=\;\frac{r^{2}\pi -\left(r^{2}\pi -\frac{1}{2}r^{2}\left(\alpha -sin\left(\alpha \right)\right)\right)}{r^{2}\pi -\frac{1}{2}r^{2}\left(\alpha -\sin\left(\alpha \right)\right)}=\frac{\alpha -sin\left(\alpha \right)}{2\pi -\left(\alpha -sin\left(\alpha \right)\right)},$$

(4.8) $$\varepsilon_{E\;}=\frac{A_{C}-A_{E}}{A_{E}}=\frac{r^{2}\pi -r\pi \frac{2r-x}{2}}{r\pi \frac{2r-x}{2}}=\frac{x}{2r-x}.$$

### 4.7. Statistical analysis

The data for this study were processed and visualized using Python’s matplotlib library (version 3.5.1), facilitating precise and customizable graphical representations of our findings. Each data point represents the mean ± standard deviation (*σ*), derived from three to eight independent experiments on individual slime mould specimens under each condition. This sample size was selected to ensure robust statistical power and accommodate biological variability inherent in our study subjects. Before performing statistical comparisons, we tested the data distributions for normality using the Shapiro–Wilk test to ensure that the assumptions required for subsequent tests were met. In all cases, data were confirmed to be normally distributed, validating the use of parametric testing for our analysis. Statistical analyses were carried out using the Python SciPy library (Version 1.8.0), chosen for its reliability and widespread accessibility in processing biological data. We employed Student’s unpaired *t*‐test (two-tailed) to compare the experimental groups with the control. This test was specifically selected owing to its suitability for datasets with independent samples that follow a normal distribution, allowing for accurate determination of significant differences between conditions. The significance levels were categorized and marked in the graphs as follows:

— for *p*-values ≤ 0.05, * indicates statistical significance;
— for *p*-values ≤ 0.01, ** denotes a high level of statistical significance; and
— for *p*-values ≤ 0.001, *** represents a very high level of statistical significance.

## Data Availability

Data and code are deposited in the Dryad Digital Repository [31].
